# Editorial: Harnessing chemotherapy resistance and development of novel therapeutic strategies for acute leukemia with KMT2A (MLL)-gene rearrangements

**DOI:** 10.3389/fphar.2022.977741

**Published:** 2022-08-08

**Authors:** Maria Teresa Esposito, Anna Hagström-Andersson, Ronald W. Stam, Stefania Bortoluzzi

**Affiliations:** ^1^ School of Life and Health Science, University of Roehampton, London, United Kingdom; ^2^ Division of Clinical Genetics, Department of Laboratory Medicine, Lund University, Lund, Sweden; ^3^ Princess Maxima Center for Pediatric Oncology, Utrecht, Netherlands; ^4^ Department of Molecular Medicine, University of Padova, Padua, Italy

**Keywords:** KMT2A, MLL, ALL, AML, Leukemia, drug repositioining

Despite the progress in the research and the availability of novel targeted therapeutic treatments to patients affected by acute leukemias, those diagnosed with chromosomal translocations affecting the *KMT2A* gene (also known as *MLL*) still face a very poor prognosis, owing to the aggressiveness and chemo-refractoriness of this leukemia. Persistence in the bone marrow of self-renewing leukemic stem cells, acquisition of secondary mutations, aberrant regulation of cell signaling, cell cycle and DNA damage repair are among the mechanisms likely to contribute to the chemo-refractory phenotype of *KMT2A-*rearranged (KMT2A-R) leukemia. In the Research Topic, “Harnessing Chemotherapy Resistance and development of Novel Therapeutic strategies for Acute Leukemia with *KMT2A (MLL)*-gene rearrangements” the authors join efforts to facilitate the emergence of new diagnostic and therapeutic strategies and review the current state of knowledge on drug repositioning to target KMT2A-R leukemia.

Drug discovery has the potential to identify brand new treatments, but the journey of these new agents from pre-clinical research to clinical trials and approval is long and the risk of clinical failure for any new compounds identified is high, due to potential adverse and unpredicted pharmacokinetic or toxicity. Drug repositioning, as reviewed by Tsakaneli et al., can lead to the identification of a candidate drug that is already known to be safe to use in humans, giving a notable acceleration to its clinical approval for a distinct disease, such as KMT2A-R leukemia. This review discusses the pre-clinical results obtained with a wide range of FDA approved drugs which have been identified to inhibit key targets in the KMT2A-epigenetic complex and signaling pathways, such as Loperamide (antidiarrheal drug), Rabeprazole (proton pump inhibitor) and Chidamide (HDAC inhibitor), which inhibit key targets in the KMT2A-epigenetic complex and downstream signaling pathway. *The Systematic In Vitro Evaluation of a Library of Approved and Pharmacologically Active Compounds”* presented by Karsa et al. is a good experimental approach for drug repositioning. With a high throughput screening of 3,707 approved drugs and pharmacologically active compounds, they identify SID7969543, described to target the transcription factor NR5A1, as a selective inhibitor of KMT2A-R cells. Interestingly, siRNA-mediated silencing of NR5A1 expression did not impact the viability of KMT2A-R cells, suggesting that the action of SID7969543 on these cells might be due to an off-target effect. This study highlights the importance of target validation and of structure-activity relationship analyses for the identification of true therapeutic targets of new drugs and for developing clinically viable inhibitors. A similar experimental approach can be employed to identify synergistic interactions between two drugs, as presented in two other studies published within this research topic. Tregnago et al. identified compounds that synergize with the Bcl2 inhibitor Venetoclax to target KMT2A-R pediatric Acute Myeloid Leukemia (AML). The study focused on leukemia with *KMT2A::MLLT3*, the most common *KMT2A*-fusion gene in AML, or with the high-risk *KMT2A::AFDN* fusion. In the latter, representing a very aggressive subtype of pediatric AML, the combination of Venetoclax and Thioridazine, an antipsychotic, resulted in an effective mitochondrial apoptotic network activation, inciting new preclinical studies. Xiao et al. identified a synergistic interaction between the nucleosome destabilizing drugs CBL0137 and the HDAC inhibitor Panobinostat in a murine model of aggressive AML driven by *KMT2A::MLLT*3 and an NRas^G12D^ mutation. Tests conducted in a xenograft model derived from an infant leukemia with the *KMT2A::MLLT1* rearrangement confirmed that the drug combination significantly prolonged survival, as compared to each drug alone. The authors further demonstrated in *vitro* models that the enhanced antileukemic effect could be obtained also using the HDAC inhibitor Entinostat, suggesting that inhibition of histone deacetylation is an important mediator of the antileukemic effect.

With a distinct approach, based on machine learning, Lopes et al. identify informative markers for prediction of *KMT2A* rearrangements in a diverse spectrum of acute leukemias and novel therapeutic options for KMT2A-R-leukemia. A small set of genes with expression highly correlated with KMT2A-R was identified as a new diagnostic model. The model integrated gene expression and clinical variables and was then validated in independent clinical subset. *SKIDA1* and *LAMP5* overexpression strongly associated with KMT2A-R and, notably, high expression of *LAMP5* could identify the cytogenetically cryptic *KMT2A::USP2* fusions that can be overlooked by Fluorescence *in situ* hybridization analysis. Moreover, using data available in the GDSC database they identified compounds effective for therapy of KMT2A-R leukemia, regardless of the leukemia subtype and proposed Foretinib, an oral multikinase inhibitor, as the most promising therapeutic candidate.

Overall the studies published in this special issue witness the unfolding of novel experimental methods that are complementing the standard approaches for target identification and validation for drug discovery. As novel bioinformatic tools have become available, new candidate drugs, including anti-helminths, antidiarrheal drugs and antipsychotic agents have been identified as potential therapeutic strategies for KMT2A-R leukemia. Further pre-clinical and clinical studies will be needed to determine the safety and efficacy of these novel treatments in patients with KMT2A-R leukemia.

